# Knowledge and Awareness of Congenital Cytomegalovirus Among Women

**DOI:** 10.1155/IDOG/2006/80383

**Published:** 2006-12-28

**Authors:** Jiyeon Jeon, Marcia Victor, Stuart P. Adler, Abigail Arwady, Gail Demmler, Karen Fowler, Johanna Goldfarb, Harry Keyserling, Mehran Massoudi, Kristin Richards, Stephanie A. S. Staras, Michael J. Cannon

**Affiliations:** ^1^National Center for Infectious Diseases, Centers for Disease Control and Prevention, Atlanta, GA 30333, USA; ^2^Department of Epidemiology, Rollins School of Public Health, Emory University, Atlanta, GA 30322, USA; ^3^National Center on Birth Defects and Developmental Disabilities, Centers for Disease Control and Prevention, Atlanta, GA 30333, USA; ^4^Department of Pediatrics, Medical College of Virginia, Virginia Commonwealth University, Richmond, VA 23298, USA; ^5^Developmental Cognitive Neuroscience Laboratory, Department of Communication Sciences and Disorders, Northwestern University, Evanston, IL 60208, USA; ^6^Department of Pediatrics, Baylor College of Medicine, Houston, TX 77030, USA; ^7^Department of Pediatrics, School of Medicine, University of Alabama at Birmingham, Birmingham, AL 35213, USA; ^8^Children’s Hospital, Cleveland Clinic, Cleveland, OH 44104, USA; ^9^Department of Pediatrics, School of Medicine, Emory University, Atlanta, GA 30322, USA

## Abstract

*Background*. Congenital cytomegalovirus (CMV) infection is a leading cause of disabilities in children, yet the general public appears to have little awareness of CMV. *Methods*. Women were surveyed about newborn infections at 7 different geographic locations. *Results*. Of the 643 women surveyed, 142 (22%) had heard of congenital CMV. Awareness increased with increasing levels of education (*P* < .0001). Women who had worked as a healthcare professional had a higher prevalence of awareness of CMV than had other women (56% versus 16%, *P* < .0001). Women who were aware of CMV were most likely to have heard about it from a healthcare provider (54%), but most could not correctly identify modes of CMV transmission or prevention. Among common causes of birth defects and childhood illnesses, women's awareness of CMV ranked last. *Conclusion*. Despite its large public health burden, few women had heard of congenital CMV, and even fewer were aware of prevention strategies.

## BACKGROUND

CMV is the most common congenital infection in the United States, affecting as many as 40 000 newborns each year [1]. Approximately, 10% of these infants are symptomatic at birth and most of these will suffer permanent neurologic sequelae such as neurodevelopmental delays, motor disabilities, deafness, and blindness [2–5]. Although most infants appear asymptomatic at birth, 10% to 15% of them will develop progressive hearing loss [6]. More children are affected by congenital CMV-related disabilities than by other, better-known childhood diseases, and syndromes, such as fetal alcohol syndrome, Down's syndrome, and neural tube defects [2, 7].

Most congenital CMV infections and related disabilities result from primary (ie, first-time) infections in pregnant women [8]. Many maternal CMV infections might be prevented by simple hygienic precautions, such as frequent and thorough hand washing [7, 9]. Despite this opportunity to prevent infection in mothers and subsequent disability in their children, anecdotal evidence suggests that the general public has little awareness or knowledge of CMV. To further investigate this evidence, and to improve the knowledge base for developing effective interventions, we designed a survey to evaluate awareness and knowledge of CMV among women.

## METHODS

We conducted a survey of congenital CMV awareness at 7 different geographic locations: Atlanta, GA; Birmingham, AL; Cleveland, OH; Provo, UT; Richmond, VA; Chicago, IL; and Houston, TX. At the first four sites, women were recruited from pediatric outpatient clinic waiting rooms. In Richmond, women were recruited from an obstetrics/gynecology clinic. In Chicago, women were recruited from a university's student union center. In Houston, the women who participated in the survey were medical students and support staff in a hospital. At each site, we asked all women aged ≥ 18 years, who were literate in English or Spanish, to complete the short, self-administered written survey. Women were told that the survey would assess their knowledge of newborn infections but not that it was designed specifically to assess knowledge of congenital CMV. The survey assessed demographic information, awareness of CMV, risk factors, modes of transmission, prevention of CMV infection, and symptoms of CMV disease. The survey also collected information about healthcare and prenatal visits, parenting behaviors, and awareness of other birth defects and childhood illnesses. When the women returned their completed surveys, they were given a brochure about congenital CMV. The study was approved by the human subjects review boards of the institutions involved.

## RESULTS

### CMV awareness

**Figure 1:**
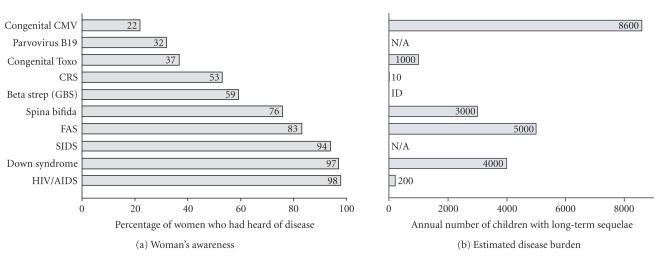
(a) Awareness of congenital CMV and other birth defects and childhood illnesses. (b) Estimates of the annual burden of prominent childhood diseases and syndromes in the US (from [7]). Assumes 4 million live births per year and 20 million children <5 years of age. Childhood deaths were defined as those occurring <1 year after birth except for *Haemophilus influenzae* type B (Hib) (<5 years) and HIV/AIDS (<13 years). Where applicable, numbers represent means of published estimates. All estimates should be considered useful for rough comparisons only since surveillance methodology and diagnostic accuracy varied over different studies. CMV: cytomegalovirus; Toxo: toxoplasmosis; CRS: congenital rubella syndrome; GBS: group B strep; FAS: fetal alcohol syndrome; SIDS: sudden infant death syndrome; N/A: not applicable because long-term sequelae are not normally associated with the condition; ID: insufficient data.

We aimed to survey approximately 100 women at each of the study sites (Table 1). Of the 643 women surveyed, 142 (22%) had heard of congenital CMV. Among other birth defects and childhood illnesses that were included in the survey, women's awareness of congenital CMV ranked last (Figure 1(a)).

In univariate analyses, awareness of CMV increased with higher levels of education (*P*-value for trend < .0001), older age (*P*-value for trend = .02), and varied by study site, with awareness being highest in Houston and Birmingham and lowest in Chicago (Table 1). In addition, women who were currently or previously employed in healthcare professions were more likely to have heard of CMV than those who had never worked in the health field (56% versus 16%, OR = 6.7 [4.3−10.6]). On the other hand, there was no significant difference in awareness of CMV between women who were currently or previously employed as a daycare worker. There was also no significant difference in CMV awareness between women who had never been pregnant and women who had been pregnant. There were no significant differences in awareness of CMV by income or race/ethnicity. After adjusting for other covariates in multivariate analyses, awareness was still associated with higher level of education and having been a healthcare professional, but was no longer associated with older age (Table 2).

*CMV knowledge among women who had heard of CMV*

Among the 142 (22%) women who had heard of CMV, there was a low level of accurate knowledge about CMV. The majority of these respondents could not correctly identify the symptoms associated with congenital CMV disease (Table 3). Approximately, one third (36%, 48/133) also indicated that congenital CMV could cause congenital heart defect, a symptom not associated with congenital CMV.

Although women who had heard of CMV appeared to lack knowledge about CMV and its symptoms, most women indicated (78%, 109/140) that a pregnant woman could pass CMV to her unborn baby. More than half (57%, 78/138) indicated that the spread of CMV could be reduced by frequent hand washing. However, nearly one quarter (23%, 83/137) incorrectly believed that CMV could be prevented by avoiding cleaning cat litter boxes. Concerning source of knowledge, 54% of women indicated that they had heard about congenital CMV from a healthcare professional (Table 4).

## DISCUSSION

To our knowledge, this was the first survey to examine women's knowledge of congenital CMV. Of the women surveyed, only 22% had heard of congenital CMV and very few had specific knowledge about clinical symptoms, modes of transmission, or prevention. There was a tremendous gap between CMV knowledge and congenital CMV disease burden (Figure 1)—women's awareness of CMV ranked last among other birth defects and common childhood illnesses despite CMV being one of the most common and most serious causes of birth defects and disabilities [7].

In order to bridge this gap, women should be educated about congenital CMV. Our survey indicated that healthcare professionals play the most important role in informing women about CMV (Table 4). Yet, only a little over half of women who were working or had worked in the healthcare field were aware of congenital CMV. Women did not specify what type of healthcare work they had done, and many might have done work that required no CMV-related knowledge or training. Nevertheless, the lack of awareness among healthcare professionals shows that there is considerable room for improvement. The curricula of medical, nursing, and midwifery schools should emphasize the importance of educating pregnant women about congenital CMV prevention through improved hand hygiene, as recommended by the American College of Obstetrics and Gynecology (ACOG) [10]. Surveys should be conducted of healthcare providers' knowledge and practices relating to congenital CMV, including how well providers adhere to these ACOG recommendations.

The survey revealed that women who were currently working or had worked in daycare centers were no more aware of congenital CMV than those who had never worked in a daycare setting. Prevalence of CMV in daycare centers is relatively high due to horizontal spread [11], and women who work with children in this setting have a higher risk of acquiring CMV infection. Day care workers should be informed of the risk of acquiring CMV infection and possible effects on the unborn child, as well as strategies for reducing risk of infection.

Many women also indicated that they were informed about CMV through school or class. Information about CMV should be included in secondary-school health curricula and in childbirth courses for expecting parents. Although educational messages need to target all women, a special effort should be made to reach women of lower educational levels, since their awareness was the lowest.

It is important that books and magazines—another key source of CMV information identified by our survey—contain better CMV information than is currently available. Many pregnancy books contain little information on CMV, and sometimes their information is inaccurate. For example, although studies show relatively high rates of transmission from young children to parents [12], one popular pregnancy book states that *“Pregnant women with toddlers of their own need not worry about catching CMV; the possibility is extremely remote.”* Such inaccuracies might be reduced by consulting physicians who are experts on congenital CMV infection, or by including information from other reliable sources, such as ACOG [10] or the Centers for Disease Control and Prevention (CDC).

**Figure 2:**
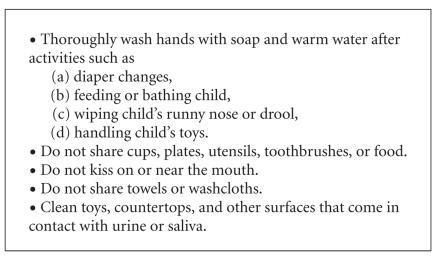
Hygienic practices to reduce risk of CMV infection for women who are pregnant or planning to become pregnant. When interacting with young children, women should assume the children are excreting CMV in their urine and saliva (from [7]).

The CMV prevention guidelines that pregnant women should receive from healthcare providers, books, magazines, websites, and other media should be straightforward and have a special focus on improved hand hygiene (Figure 2) [7]. Examples of these guidelines can be found at websites such as http://www.cdc.gov/cmv and http://www.bcm.edu/pedi/infect/cmv. Risk of CMV infection is likely to be reduced by adherence to these guidelines [7], and pregnant women have proven receptive to other behavior changes that protect their babies [9, 13–19]. In our survey, for example, nearly all women reported that during their latest pregnancy, they took prenatal vitamins on a regular basis (90%) and did not drink alcohol (94%). Further research is needed to assess which prevention messages work best for CMV and which educational interventions are most effective.

Our survey was subject to several limitations. The questionnaire was not pretested to eliminate ambiguous questions, and some of the respondents did not follow directions when answering questions. Another limitation was that the survey was a convenience sample that may not represent all women in the US. This type of sampling led to significant variation in awareness at different sites. For example, the lower awareness in Chicago was probably due to the women being young, college students, whereas the higher awareness in Houston was probably due to the women being medical students and hospital support staff. Nevertheless, the seven different geographical locations included a diverse population of women, so the results should be reasonably applicable to women of childbearing age. Another study limitation was that guessing may have played a role in answering specific knowledge questions about CMV. For example, many women incorrectly answered that CMV can cause congenital heart defects. This implies that some women may have guessed correctly about other questions. Hence, our survey may overestimate women's knowledge about CMV. Selection bias may also have affected our results. The majority of the surveys were administered in a clinic setting, and women attending a healthcare clinic may be more likely to be aware of health issues than other women. Such bias may have led to overestimates of women's awareness of CMV.

Raising awareness among women and their healthcare providers will be an important first step for preventing congenital CMV. Such awareness can lead to improvement in hygiene behaviors among pregnant women, immediately impacting the congenital CMV disease burden. Furthermore, as women and their healthcare providers become educated about congenital CMV, they will better appreciate the potential for interventions such as prenatal screening and diagnosis [20], newborn screening [21], and antiviral [22] or hyperimmuneglobulin treatments [23], and they will see the urgent need for the development of an effective CMV vaccine [24].

## Figures and Tables

**Table 1:** Awareness of congenital CMV by characteristics of women. Note that not all questions had responses from all participants.

Characteristic	Aware of CMV		Unaware of CMV		Odds ratio	95% CI^(a)^	*P*-value^(b)^
	*n* = 142	Percentage	*n* = 497	Percentage			

Site
Chicago, IL (reference)	8	7	100	93	1
Provo, UT	5	12	38	88	1.6	(0.5–5.3)	.41
Richmond, VA	16	18	73	82	2.7	(1.1–6.7)	.02
Cleveland, OH	21	21	79	79	3.3	(1.4–7.9)	.005
Atlanta, GA	28	26	81	74	4.3	(1.9–10.0)	.0003
Birmingham, AL	26	30	62	70	5.2	(2.2–12.3)	< .0001
Houston, TX	38	37	64	63	7.4	(3.3–16.9)	< .0001
Race
Whites (reference)	82	23	278	77	1
Blacks	39	22	137	78	1.0	(0.6–1.5)	.87
Hispanics	12	24	37	76	1.1	(0.6–2.2)	.79
Asians	6	16	31	84	0.7	(0.3–1.6)	.36
Other	3	19	13	81	0.8	(0.2–2.8)	.71
Age
Under 20 (reference)	4	15	22	85	1		.02
20–29	64	21	245	79	1.4	(0.5–4.3)
30–39	41	22	147	78	1.5	(0.5–4.7)
40–49	25	27	69	73	2.0	(0.6–6.4)
Over 49	8	47	9	53	4.9	(1.2–20.4)
Level of education
Less than high school (reference)	1	4	24	96	1		< .0001
High-school diploma or GED	33	17	166	83	4.8	(0.6–36.5)
Some college	43	21	158	79	6.5	(0.9–49.7)
bachelor's degree or more	65	31	145	69	10.8	(1.4–81.2)
Level of income
Less than $20 000 (reference)	28	24	91	76	1		.62
$20 000–49 999	42	24	131	76	1.0	(0.6–1.8)
$50 000–74 999	25	21	94	79	0.9	(0.5–1.6)
$75 000–100 000	21	25	62	75	1.1	(0.6–2.1)
More than $100 000	23	21	89	79	0.8	(0.5–1.6)
Ever worked as a healthcare professional
No (reference)	83	16	443	84	1		< .0001
Yes	58	56	46	44	6.7	(4.3–10.6)
Ever worked as a daycare worker
No (reference)	108	21	405	79	1		.18
Yes	32	27	88	73	1.4	(0.9–2.2)
Location where healthcare received
private doctor or HMO (reference)	89	22	310	78	1		.81
Other	53	23	176	77	1.1	(0.7–1.5)
Ever been pregnant
No (reference)	39	19	166	81	1		.15
Yes	103	24	321	76	1.4	(0.9–2.1)

^(a)^CI: confidence interval.

^(b)^ *P*-value of association or trend.

**Table 2:** Logistic regression summary: awareness of congenital CMV among women. Some categories were collapsed in the multivariate analysis because of small numbers in the categories.

Characteristic	Odds ratio	95% CI^(a)^	*P*-value^(b)^

Site			
Chicago, IL (reference)	1	—	.58
Provo, UT	1.4	(0.3–6.7)	
Richmond, VA	4.6	(1.2–16.7)	
Cleveland, OH	2.3	(0.6–8.2)	
Atlanta, GA	4.4	(1.3–15.6)	
Birmingham, AL	7.6	(1.9–30.6)	
Houston, TX	5.3	(1.8–15.8)	
Race			
Whites (reference)	1	—	.66
Blacks	0.8	(0.4–1.5)	
Hispanics	0.9	(0.4–2.1)	
Other	1.1	(0.4–2.9)	
Age			
Under 30 (reference)	1	—	.90
30–39	0.6	(0.3-1.0)	
40 and over	1.0	(0.5–1.9)	
Level of education			
High-school diploma or less	1	—	.03
Some college	1.5	(0.8–2.7)	
bachelor's degree or more	2.1	(1.1–4.1)	
Level of income			
less than $20 000 (reference)	1	—	.82
$20 000–49 999	1.1	(0.6–2.1)	
$50 000–74 999	0.9	(0.4–1.9)	
$75 000–100 000	1.3	(0.5–3.0)	
More than $100 000	0.9	(0.4–2.1)	
Ever worked as a healthcare professional			
No (reference)	1	—	< .0001
Yes	6.8	(3.9–11.7)	
Ever worked as a daycare worker			
No (reference)	1	—	.99
Yes	0.9	(0.5–1.6)	
Location where healthcare received			
private doctor or HMO (reference)	1	—	.97
Other	1.0	(0.6–1.7)	
Ever been pregnant			
No (reference)	1	—	.39
Yes	1.2	(0.5–2.5)	

^(a)^ CI: confidence interval.

^(b)^ *P*-value of association or trend.

**Table 3:** Percentage of women responding to questions regarding which of the following clinical manifestations were caused by congenital CMV in newborns. Note that these figures are among the 22% of the women who have heard of CMV.

Congenital CMV in newborns can cause	Yes (%)	No (%)	Do not know (%)

Hearing loss	48*	8	44
Mental retardation	47*	5	48
Jaundice	24*	13	63
Seizures	34*	6	60
Microcephaly	30*	8	63
Death	35*	7	58
Club foot	8	21*	71
Congenital heart defect	36	7*	57

*Correct answers.

**Table 4:** Source of CMV awareness or knowledge. Note that multiple answers were accepted so the percentages add to more than 100%.

Source	Number	Percentage

Healthcare provider	79	54
School or class	46	32
Magazine, book, or newspaper	23	16
Family or friends	17	12
Other	14	10
Internet	7	5
Radio or TV	5	3
